# Reducing biomass burning is key to decrease PM_2.5_ exposure in European cities

**DOI:** 10.1038/s41598-024-60946-2

**Published:** 2024-05-03

**Authors:** Stefano Zauli-Sajani, Philippe Thunis, Enrico Pisoni, Bertrand Bessagnet, Fabio Monforti-Ferrario, Alexander De Meij, Ferenc Pekar, Elisabetta Vignati

**Affiliations:** 1 European Commission, Joint Research Centre, Ispra, VA Italy; 2 MetClim, Varese, Italy

**Keywords:** Urban PM_2.5_ pollution, Sources of air pollution, Residential sector, Biomass burning, Air quality and climate policies, Environmental impact, Climate-change mitigation, Risk factors

## Abstract

Throughout the world, ambient fine particulate matter (PM_2.5_) is the environmental factor that poses the greatest risk to health and most European citizens continue to be exposed to PM_2.5_ levels well above World Health Organization guidelines. Here we present a comprehensive PM_2.5_ modelling-based source allocation assessment in 708 urban areas in Europe. The results show that urban cores, together with their commuting zones, contribute an average of 22% to urban PM_2.5_ concentrations levels. The residential sector is the highest source sector in 56% of cities. Its average contribution to PM_2.5_ formation is 27%, with a cluster of cities in Northern Italy and Eastern Europe contributing to more than 50%. Industry, agriculture and road transport show average contributions of 18%, 17% and 14%, respectively. Most emissions from residential sectors are anthropogenic primary PM_2.5_ which includes a condensable fraction. Furthermore, anthropogenic primary PM_2.5_ represents the precursor with the highest contribution in most cities (72%), contributing an average of 35% to urban PM_2.5_ levels. Emissions of anthropogenic primary PM_2.5_ by the residential sector are almost entirely (with exceptions of few countries) due to biomass burning. These results suggest that the residential sector should be a key target of any policy to improve air quality and that climate policies promoting biomass as a climate-neutral fuel could have a detrimental effect on air quality. A more integrated approach to climate and air quality policy design is desirable.

## Introduction

Exposure to air pollutants has been proven to have a range of detrimental health effects, including premature mortality from cardiovascular and respiratory disease and cancer^1^. Strong evidence of the health effects has been provided in particular for fine particulate matter (PM_2.5_) which has been indicated as the largest environmental risk for human health^2^.

In Europe, the application of air quality policies, together with technological development, has led to substantial improvements in recent decades. However, in large European areas, the PM_2.5_ level remains well above the World Health Organization (WHO) guideline value^3^ and frequently exceeds the current EU limit^4^. In 2022, the European Environment Agency estimated that 96% of the urban population of the European Union (EU) is exposed to concentrations of PM_2.5_ above the WHO guideline of 5 µg/m^3^^5^ and that exposure to fine particulate matter accounts for approximately 238.000 premature deaths in the EU-27 each year.

The wide range of human activities that contribute, directly or indirectly, to PM_2.5_ formation and the complexity of the physical and chemical processes involved in its transformation^6,7^ make particularly challenging to design mitigation strategies and air quality plans. Chemistry transport models (CTM) can be very useful for this purpose, as they can simulate the complex interactions that occur between gases, as well as liquid and solid particles, and help identify the role of the different sources. Unfortunately, these models require not only a high level of professional competence but also intensive computational resources. To overcome these limitations, and to help local authorities to develop tailored air quality plans based on scientific data, the Joint Research Centre of the European Commission developed the Screening for High Emission Reduction Potential for Air Quality tool (Sherpa). This tool mimics a CTM with much shorter computation time, and produces results of similar accuracy when air pollutant concentrations are considered over long time periods (seasonal or annual averages)^8^. More details of the Sherpa tool are given in Section “Methods”.

In a previous work^9^, Sherpa was used to assess the sources of urban PM_2.5_ in terms of different spatial scales, emission sectors and precursors in 150 major EU cities.

This study represents an evolution of the previous studies in various respects. In particular, key novelty aspects of this paper (in comparison to previous papers, and to the scientific literature) lie in the following aspects: (a) the number of cities analyzed in a harmonized way has increased to more than 700; (b) the model spatial resolution is at 6 km, that is already quite high for a Europe-wide study; (c) the period covered by the input data is 2019, that is to say the most recent (pre-pandemic) available year. Finally, (d) for the first time the analysis includes the emission from condensables in primary PM emissions (important to describe residential sector emissions), and an improved differential treatment of high-level and surface emissions (to increase the accuracy of the results).

The focus in this work is on the residential sector and, in particular, biomass burning. The relevance of the contribution from the residential sector has been documented by a number of studies^10,11^, and in recent years residential emissions have gained further attention because they can be considered from multiple perspectives, including indoor and outdoor air quality, energy production, climate policies. This may require trade-offs because actions that are beneficial in one respect may have harmful effects in other areas. Particularly interesting is the theme of biomass burning which has been suggested as a climate-friendly solution for domestic heating but which can worsen air quality and human health^12^.

## Results

### Spatial source allocation

Understanding the origin of urban pollution is key to shaping air quality plans and define effective air pollution mitigation actions. This topic (here called spatial source allocation) is analyzed in terms of three spatial aggregations of administrative entities: city core, Functional Urban Areas (FUA, city core together with its commuting zone—see Section “Methods”) and country. A total of 708 urban areas in the EU-27, Norway, Switzerland and the United Kingdom were included in the analysis, together accounting for 64% of the total population of the study area.

**Figure 1:**
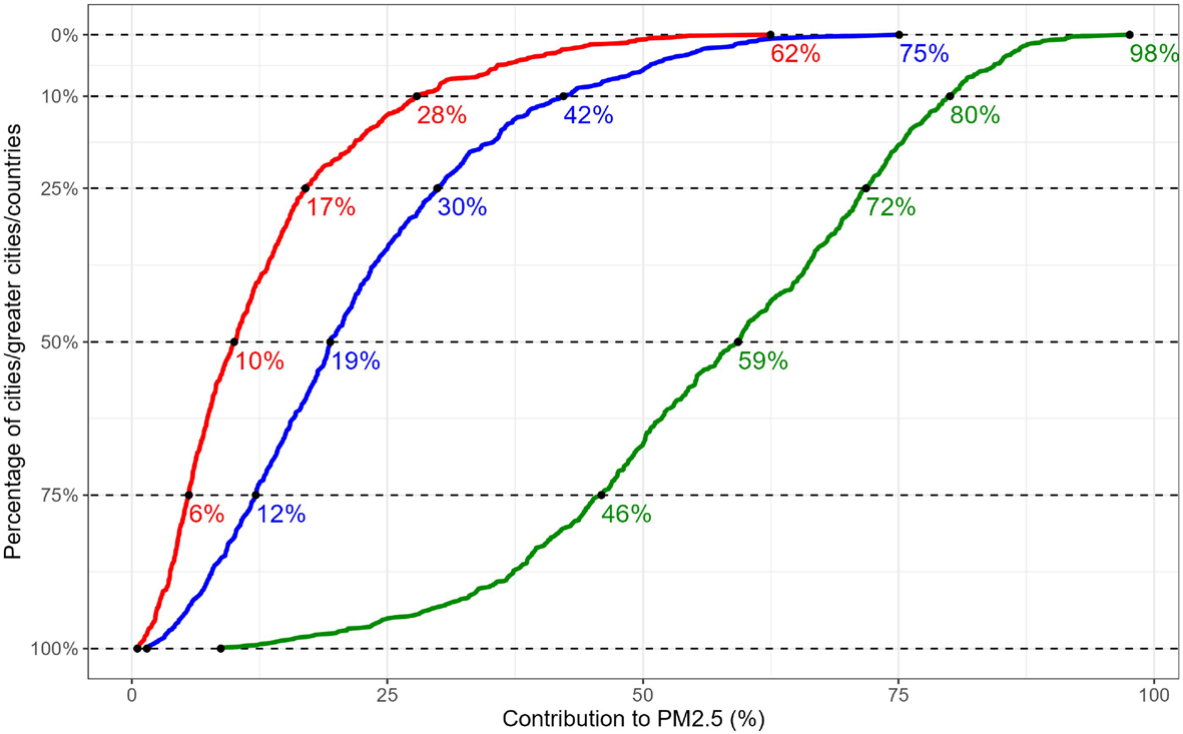
Contribution of emissions from city, FUA and country to PM_2.5_ concentrations. Cumulative frequency distribution showing the percentage of cities/FUAs/countries (respectively in Figure in red, blue and green) contributing more than a given percentage to the PM_2.5_ urban concentration. The contributions corresponding 100%, 75%, 50%, 25%, 10%, and 0% (i.e. maximum contribution among cities/greater cities and countries) are highlighted.

Cities’ contribution to annual urban PM_2.5_ concentrations ranges from 1 to 62% with a mean value of 13%, but 25% of cities contribute more than 17%, and 10% contribute more than 28% (Fig. 1). Including commuting zones in the assessment of the cities’ contributions (i.e. considering FUAs) substantially increases the contributions of urban areas. The mean contribution from FUAs is 22% with the highest values found in Oslo (75%), Warsaw (72%), Lisbon (68%), Paris (65%), Madrid (63%) and the Ruhr area (62%). Overall, 25% of FUAs account for more than 30% to PM_2.5_ and 10% of FUAs account for more than 42%.

The population of cities and FUAs is an important predictor of their contribution to urban PM_2.5_ concentration. Figure 7 (supplementary material) shows the relationship between city and FUA population and their contribution to PM_2.5_.

On average (over all 708 cities), the share of local PM_2.5_ that is attributable to emissions from the entire country is around 58%. In a quarter of the cities, the country accounts for at least 75% of local PM_2.5_, while in 10% of cities the country contribution is higher than 80%.

Spatial sources of PM_2.5_ other than the city, FUA and country include transboundary and natural transport, international shipping and other sources outside the modelling domain. Transboundary contributions are generally low except in the case of some cities close to the countries’ boundaries and/or in countries with a small area. Examples of cities heavily affected by transboundary contributions are Maastricht in the Netherlands (75%), Lugano in Switzerland (74%) and Luxembourg City in Luxembourg (68%).

### The primary role of the emissions from the residential sector

In this section we analyse the contribution to urban background PM_2.5_ concentration of several aggregations of anthropogenic and natural sources. In particular, we distinguish and quantify the contributions from the following macro-sectors: residential, transport, agriculture, industry, all other anthropogenic sectors (from now on “other”), natural and external (i.e. all emissions from outside the model domain).

**Figure 2:**
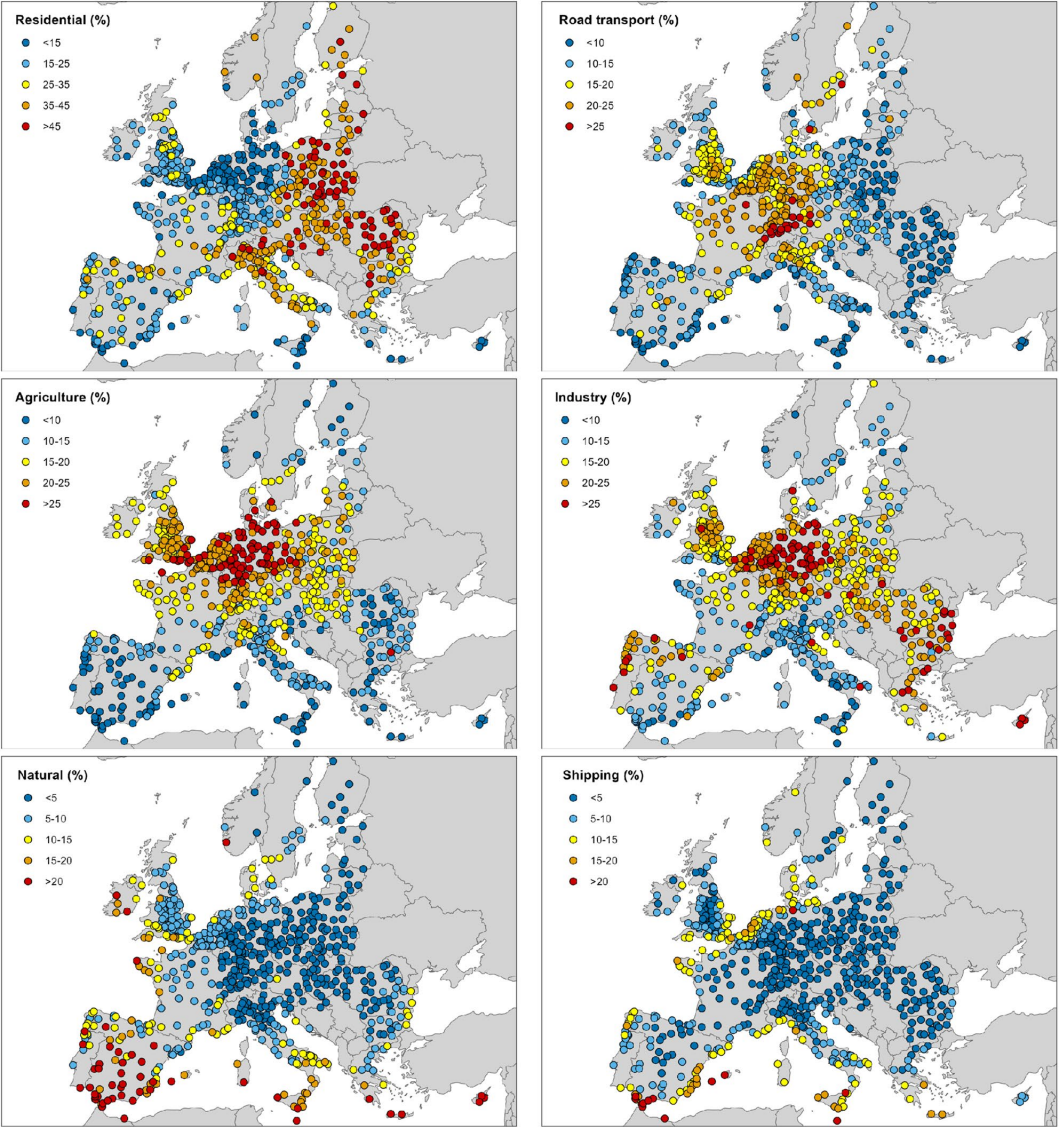
Contribution of the individual emission sectors to local PM_2.5_ concentrations. Each dot represents an urban area considered in this study.

Figure 2 provides an overview of the contribution of the different emission sectors. Sectoral contributions are the overall contribution of emissions from each sector, regardless of their spatial origin.

The residential sector is the largest contributor, accounting for, on average, 27% of local PM_2.5_ and is the highest source sector in 56% of the cities. The highest contribution from the residential sector can be observed in Poland, Romania, Northern Italy, Croatia, and the Baltic countries.

The average contribution from road transport is 14%. The contribution of this sector is highest on the northern side of the Alps and in some of the largest EU urban areas, such as Madrid (24%), London (23%) and Paris (22%). Note that only urban background concentrations are considered in this study, and concentrations and city contributions at high-traffic sites are likely to be proportionally higher.

The average contribution from agriculture is 17%. The largest contribution from this sector is observed in Germany, where in many cities the agriculture contribution is above 25%. High agriculture contributions are also found in other central and eastern European cities as well as in the United Kingdom. It is notable that, although emissions from agricultural sources are mainly concentrated in rural areas, secondary inorganic pollution associated with medium- and long-range transport means that this contribution is also very significant in urban areas.

The average contribution of industry in the selected urban areas is 18%. The largest contributions (> 30%) are found in Bulgaria, Germany, Netherlands, and Romania. High concentrations can also be observed in some isolated areas in Cyprus, Italy and Spain.

The contribution of natural sources is on average only 8% but is extremely high (> 40%) in some cities located at the southernmost latitudes. However, some northern European cities in France, Ireland and Norway, also show significant natural contribution (> 20%).

As expected, emissions from shipping have a large impact only in coastal cities. The largest contributions from shipping are found in Mediterranean cities located close to the east–west international shipping route, especially (> 30%) in cities near the Strait of Gibraltar. The contribution of emissions from outside the modelling domain is generally very low but reaches high values (more than 25%) in some southernmost cities as well as in some northern cities.

**Figure 3:**
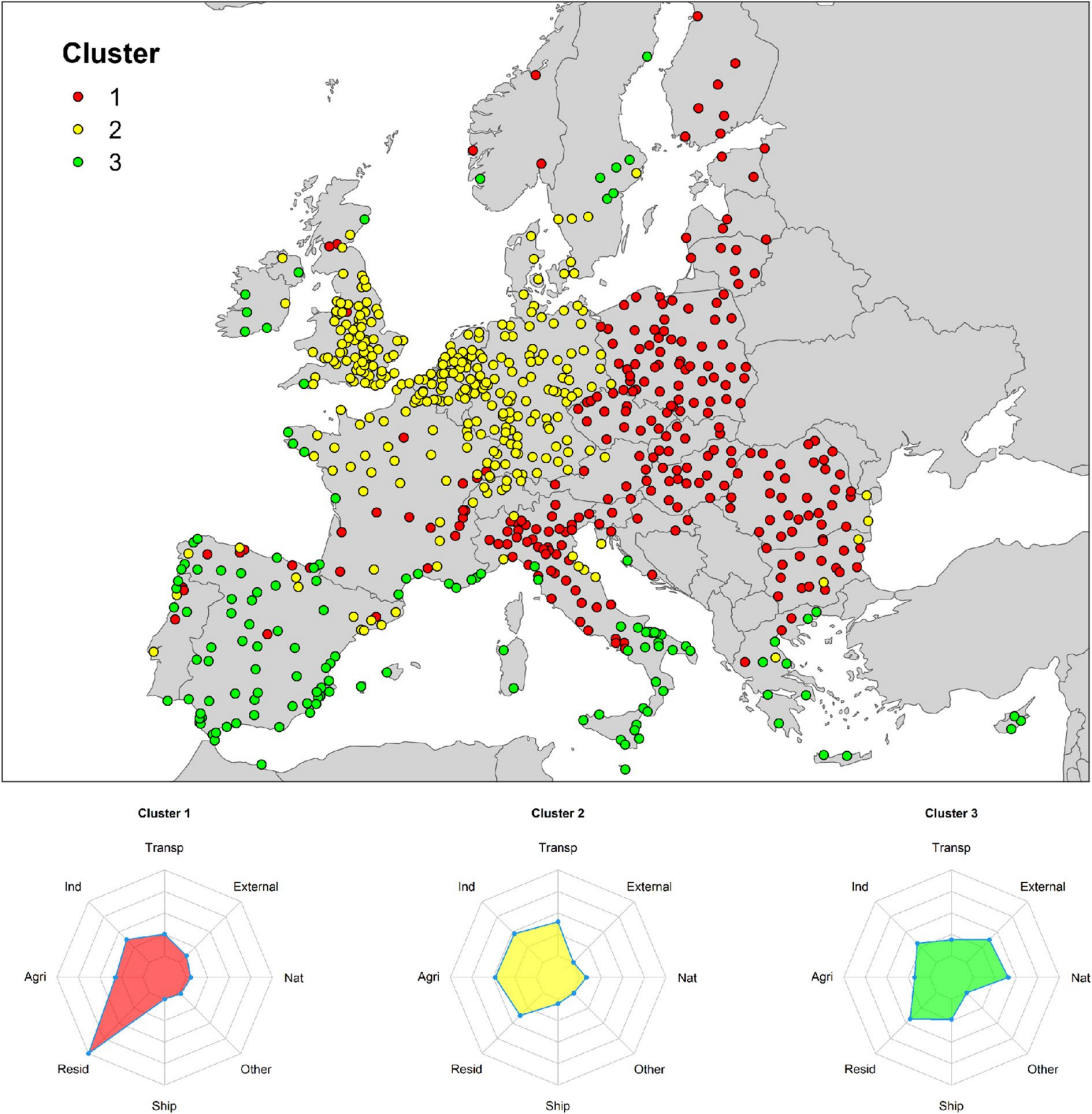
Clusters of cities by emission sectors. Map of cities clustered on the basis of percentage contributions to PM_2.5_ concentrations by sector (upper panel) and diagrams of the weight factors associated with each cluster (lower panels).

In order to identify spatial similarities among the selected cities with regards to the role of the different emission sectors, we carried out a k-means cluster analysis^13^. To find the optimal number of clusters, we chose to use the graphical ’elbow method’. The elbow graph shows the within-cluster sum-of-square values (on the y-axis) corresponding to different values of K (on the x-axis). The optimal K value is the point at which the graph forms an elbow (best option = three clusters). Figure 3 shows the well-defined spatial patterns resulting from the application of cluster analysis. One cluster (cluster 1—red dots) comprises cities where the principal source of emissions is the residential sector. This group includes many cities in Scandinavia, the Baltic countries, eastern Europe and central and northern Italy. A second cluster (cluster 2—yellow dots) comprises cities characterized by similar contributions from the main anthropogenic sources (i.e. transport, industry, agriculture and residential sectors). Cities in the cluster are mainly located in central Europe and the United Kingdom. The third cluster (cluster 3—green dots) is made up of cities where the biggest contributors are the shipping sector and the external and natural sources.

### *Anthropogenic primary PM*_2.5_* versus gaseous precursors*

**Figure 4:**
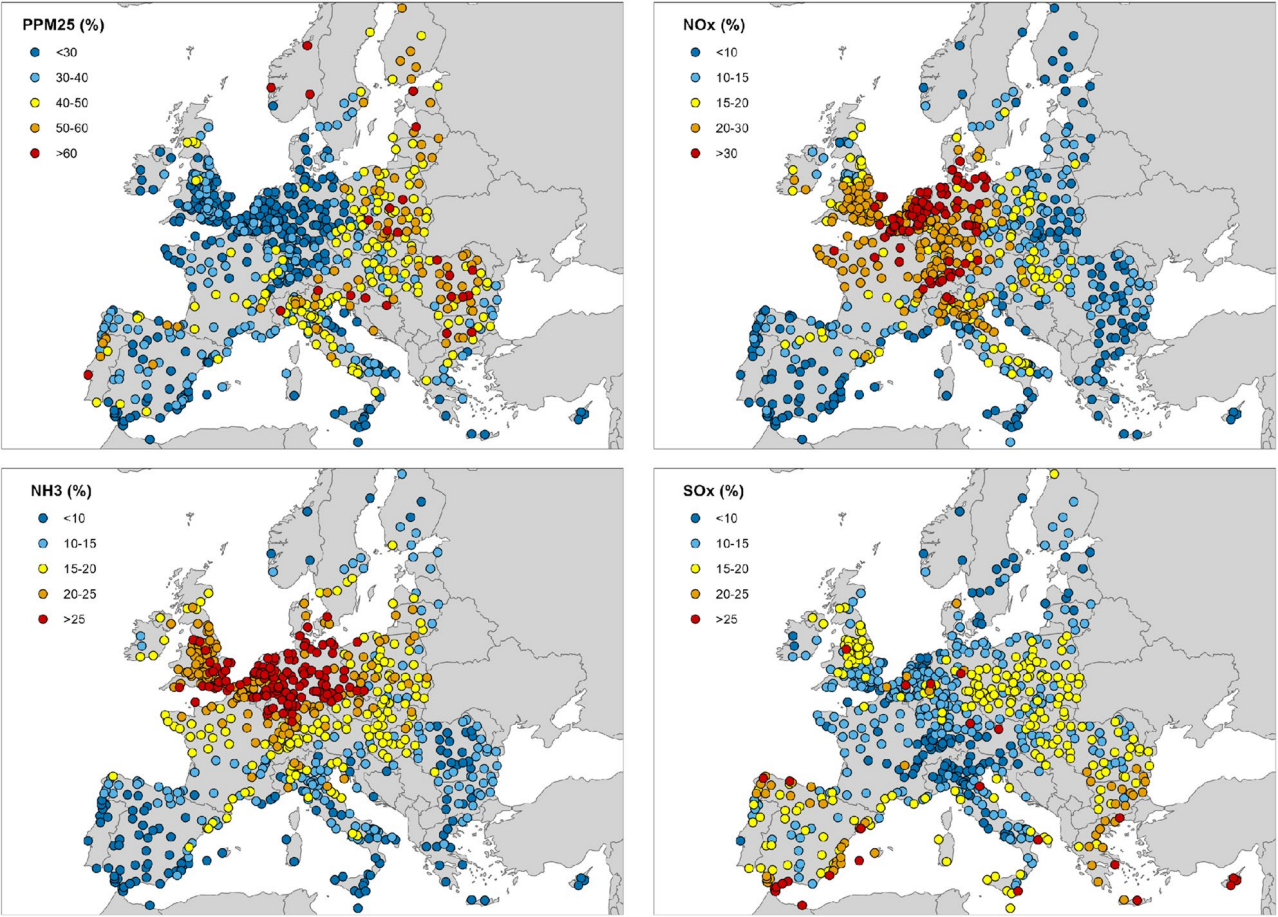
Contribution of the individual precursors to local PM_2.5_ concentrations. Each dot represents an urban area.

In this section we report the results of our analysis of the role of different precursors in determining PM_2.5_ concentrations in the selected cities. Figure 4 provides an overview across the study area of the contributions to PM_2.5_ accounted for by primary anthropogenic emissions of PM_2.5_ (anthro-PPM_2.5_), NO_*x*_, NH_3_, and SO_*x*_. In 72% of the 708 urban areas studied, the precursor that makes the greatest contribution to PM_2.5_ is anthro-PPM_2.5_, with the average contribution being 35%. The contribution of anthro-PPM_2.5_ is especially important in eastern European countries, the Scandinavian and Baltic countries, northern Italy and Portugal. Examples of cities where the contribution of anthro-PPM_2.5_ is particularly high are Lisbon in Portugal (71%), Oslo in Norway (71%), Warsaw in Poland (66%), and Turin in Italy (63%).

The average contribution of NO_*x*_ is 17%. The highest values are found in Belgium, Denmark, Northern Germany, the Netherlands and Switzerland. The average contribution of NH_3_ is 17%. The highest values are found in Belgium, Denmark, Germany, the Netherlands and the United Kingdom. The average contribution of SO_*x*_ is 15%. The contribution of SO_*x*_ is highest (> 25%) in cities characterised by the presence of important industrial plants, such as in the Ruhr area of Germany (38%), and in Ravenna and Taranto in Italy (both 27%), and in areas affected by shipping emissions.

**Figure 5:**
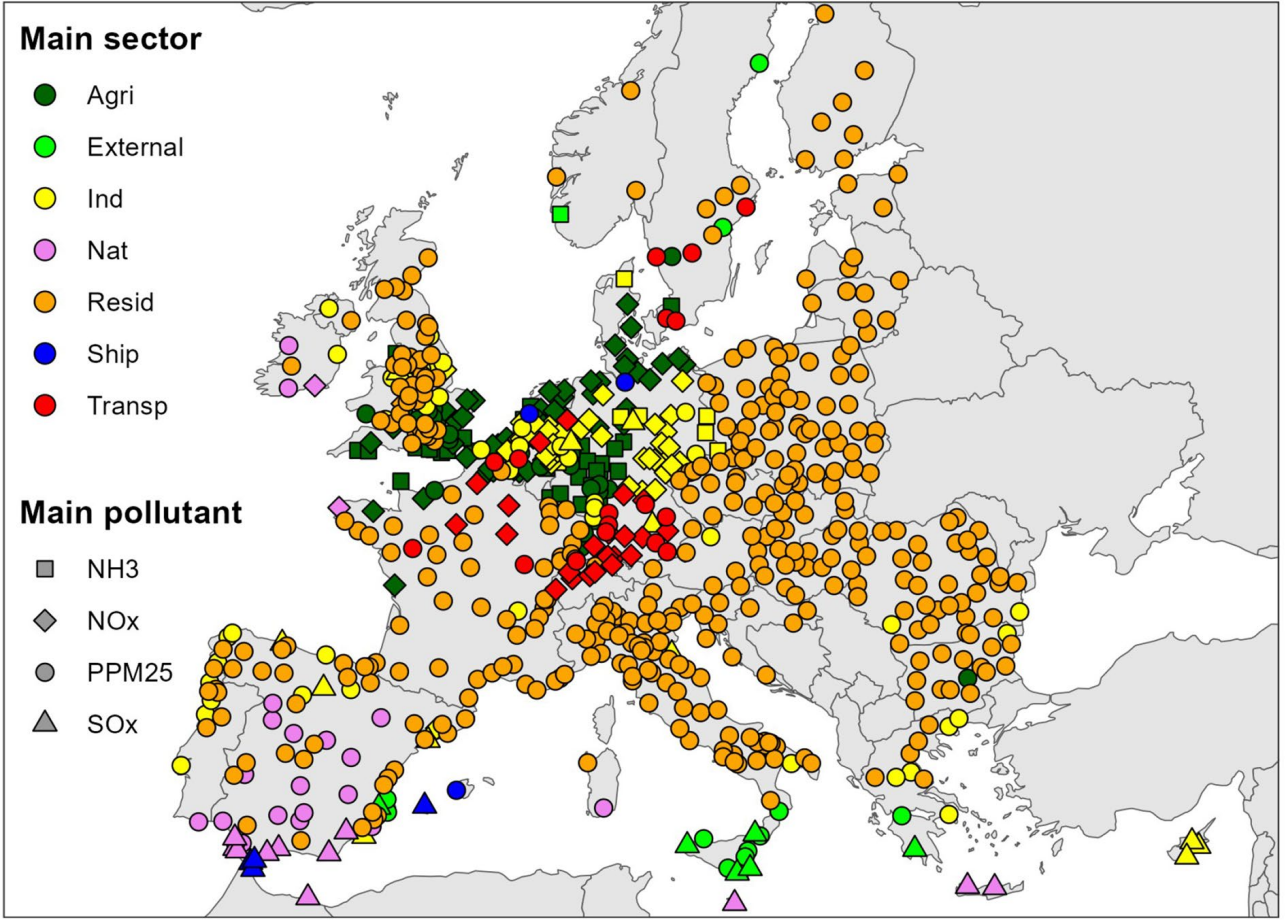
Main emission sector and precursor in each city. Sector are expressed by color, and precursor by shape, showing how they are contributing to PM_2.5_ concentrations in each city.

Figure 5 provides an overview of the main precursors and sectors accounting for PM_2.5_ in each city while Fig. 8 (supplementary material) highlights the strict link between the residential sector contribution and anthro-PPM_2.5_ emissions.

We carried out a further cluster analysis, this time focusing on the contribution of each precursor to PM_2.5_. Figure 9 (supplementary material) shows that the spatial patterns of emissions sectors and precursors identified by the cluster analyses are remarkably similar. The cluster of cities where the primary contributor is the residential sector (cluster 1 in Fig. 3) is spatially similar to the cluster of cities characterized by the fact that anthro-PPM_2.5_ is the dominant contributor (cluster 1 in Fig. 9, supplementary material). Analogously, the cluster associated with comparable contributions to PM_2.5_ formation from transport, industry, agriculture and residential sectors (cluster 2 in Fig. 3) appears spatially similar to the pollutant cluster showing equal contributions from NO_*x*_, NH_3_ and anthro-PPM_2.5_ (cluster 2 in Fig. 9, supplementary material). Finally, the cluster associated with the highest contribution to PM_2.5_ formation from the shipping sector and natural and external sources (cluster 3 in Fig. 3) shows spatial similarities with the anthro-PPM_2.5_-SO_*x*_ pollutant cluster (cluster 3 in Fig. 9, supplementary material). This shows the consistency in the presented results and provides useful information in designing mitigation strategies.

## Discussion

This study examines the contributions to PM_2.5_ provided by different spatial scales, emissions sectors and precursors, and considers two main focuses.

The first focus is on urban areas, where most of EU population (more than 70%) live^14^ and where most exposure to air pollutants takes place. The study includes all European (EU-27 plus Norway, Switzerland and the United Kingdom) urban areas with more than 50.000 inhabitants and considers urban areas in two different ways: core cities and FUAs. The core city is the part of a city characterized by the highest population density. The FUA is an extension of the core city, and is a concept developed by the Organisation for Economic Co-operation and Development (OECD) and the EU to characterize urban areas in economic and social terms, and also considers a city’s commuting zone. We suggest that air quality plans are more effective if designed at the FUA spatial scale, as an urban area’s contribution to city PM_2.5_ is almost doubled when the commuting zone is taken into account. The emission inventory used in this study is a substantial improvement on previous inventories, enabling us to understand the peculiarities of city cores and commuting zones^15^. It is important to note that the contribution from people residing in the commuting zone is especially relevant for the residential sector. Figure 10 (supplementary material) shows that per capita emissions are much higher in the commuting zone than in city core. Figures 10 and 11 (supplementary material) show that the total and per capita contributions of anthro-PPM_2.5_ emissions from the residential sector are much greater in commuting zones than in cities. Our findings thus show the importance of collaboration between nearby local administrations belonging to the same FUA (with the aim of improving air quality). Our results also suggest that promoting synergies between cities at the wider spatial scale could be beneficial, as we identified clear spatial clusters covering supranational areas in terms of emission patterns, the contributions of different precursors and mitigation priorities. These clusters could be considered to define geographical areas where cooperation between local authorities would be beneficial, enabling the creation of harmonised air quality policies targeting common PM_2.5_ emission sources and chemical regimes.

The second main focus of the study is the role of residential sector. We show that the residential sector is the main single contributor to PM_2.5_ concentrations in 56% of the cities studied. Anthro-PPM_2.5_ accounts for about 85% of the residential sector’s contribution, with NO_*x*_ and SO_*x*_ responsible for the remaining 15%. While the contribution of the residential sector to PM_2.5_ has been highlighted by several studies^16^, our results suggest that the contribution of this sector could be even greater than previously reported. In our opinion, there are three main reasons for our higher finding. First, most studies provide results averaged over large areas, including countryside, whereas our work assesses the contribution of different sources over smaller areas, comprising urban and suburban regions, where residential emissions are relatively higher. Second, in most studies, source sector analyses are based on models with considerably coarser spatial resolution than the model used in this work^12,17,18^. This is likely to affect the assessment of the source sector and may have the result of smoothing cities’ contribution to population exposure^19^. Third, in most studies the simulations are based on less recent data, where emissions patterns and assumptions were different^11,17^. Indeed, the higher contribution of the residential sector found in this study may be explained by the combined impact of the following three factors: (a) the decrease in SO_*x*_, NO_*x*_ and, to a less extent, NH_3_ emissions observed in Europe in the last decade^20^; (b) the increase in the share of the emissions of primary PM_2.5_ accounted for by the residential sector with respect to total primary PM_2.5_ emissions^20^; and (c) the inclusion in recent emission inventories of the contribution of condensable gases to primary PM_2.5_ emissions^21^. Figure 11 (supplementary material) provides city-specific estimates of PM_2.5_ contributions from the residential sector obtained by Sherpa when emissions due to condensable gases are included and not included.

Although the residential sector is the most important emissions sector, and anthro-PPM_2.5_ the most important precursor, the contribution from other emissions sectors and of secondary pollution should not be underestimated. In fact, in most cities, the relative contribution of secondary PM_2.5_ is higher than that of primary PM_2.5_ (Fig. 12, supplementary material). However, secondary pollution originates from a complex combination of emissions sectors and precursors (Figs. 13–15, supplementary material) with the possible activation of different chemical regimes also at relatively short distances^22^. This makes mitigation strategies to reduce secondary PM_2.5_ particularly important, but tricky to design and implement^7,23^. In contrast, the impact of actions targeting the residential sector and anthro-PPM_2.5_ are easier to assess, given that emissions and concentration changes are linearly related.

**Figure 6:**
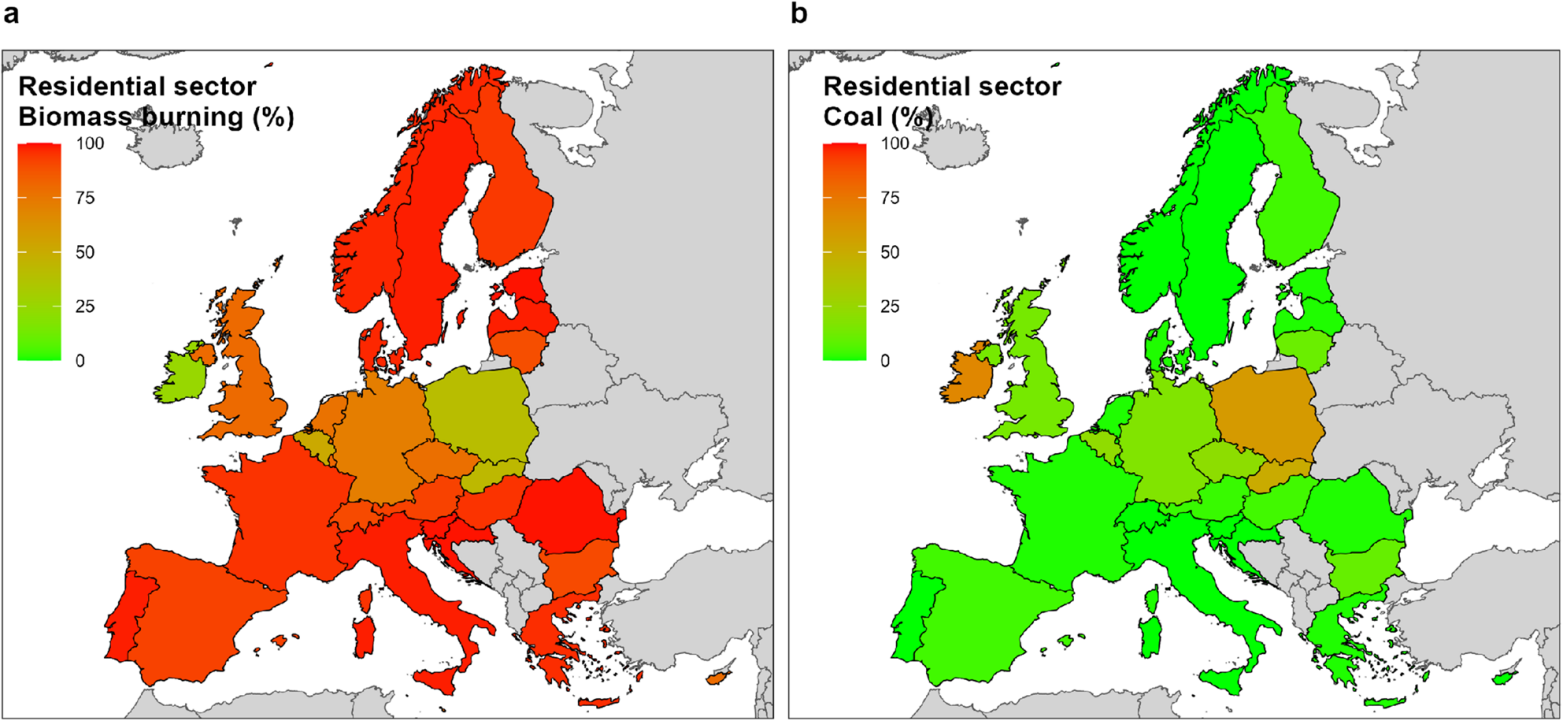
Country-specific contribution of biomass burning and coal in total emissions of PM_2.5_ from the residential sector. Percentage of primary anthropogenic PM_2.5_ (anthro-PPM_2.5_) emissions from the residential sector due to biomass burning (**a**) and coal (**b**) in the study area is shown. EDGAR data 2018.

In this study we do not consider the different type of fuels associated with the various emissions sectors. However, it is worth discussing which fuels are associated with anthro-PPM_2.5_ emissions from the residential sector. For this we rely on the Emission Database for Global Atmospheric Research (EDGAR)^24,25^. Figure 6 provides some key data. Panel a shows that, in most European countries, anthro-PPM_2.5_ from the residential sector is almost entirely emitted by biomass burning activities, while coal combustion plays a major role only in Ireland, Poland and Slovakia (panel b). Although available data show an increasing use of biomass (and woody biomass in particular, widely used for domestic heating) in the EU in the past two decades (around 20% since 2000)^26^, emission data^20^ for the same period show a slight decrease of anthro-PPM_2.5_ contributed by the residential sector. This emission trend can be largely attributed to improvements of the technologies used to burn biomass and to the progressive abandonment of the use of coal in the few European countries where it was used as a reference fuel for heating buildings. The changes in the fuel used for domestic heating observed in Europe can be attributed both to the desire to reduce costs and to policy recommendations. In fact, various policy and technical documents, including some issued by the EU, considered biomass to be a carbon neutral fuel. The transition from the use of fossil fuels to biomass for the production of energy and heat has been therefore widely supported at international level. In the EU, biomass heat increased by about 50% between 2005 and 2020 and nowadays biomass burning accounts for about 50% of all energy produced from renewable sources^27^. Interestingly, there is still a lively debate in the scientific literature about the actual potential role of biomass burning in reducing greenhouse gas (GHG) emissions. While some studies report that significant reductions in emissions can be achieved in the short term^28–30^, others have found that, over a time scale of decades or centuries, biomass burning produces more GHG emissions than the burning of fossil fuels^31–33^. In addition, European Green Deal (https://commission.europa.eu/strategy-and-policy/priorities-2019-2024/european-green-deal_en) and 2030 Biodiversity Strategy (https://environment.ec.europa.eu/strategy/biodiversity-strategy-2030_en) warn that the use of biomass to produce energy and heat may have a significant negative impact on local biodiversity and ecosystems. Therefore, to maintain an appropriate balance, in the EU, the use of biomass to produce energy has to adhere to the strict sustainability criteria defined in the Renewable Energy Directive (http://data.europa.eu/eli/dir/2023/2413/oj) and should privilege the re-use of biomass wastes and residues from both agriculture, forestry and industry sectors^34^.

Further concerns about intensive use of biomass burning come from the epidemiological and toxicological literature. The health risks associated with emissions of NO_*x*_, polycyclic aromatic hydrocarbons (in particular benzo(a)pyrene), volatile organic compounds and dioxins originating from biomass burning are well known and, while the potential of specific individual sources and chemical components of PM_2.5_ to cause adverse health outcomes is still unclear^35^, several studies have suggested that biomass burning may have a significant health impact^17,36^.

In conclusion, limiting emissions from residential sectors is key to the reduction of PM_2.5_ concentrations. Specific attention should be given to biomass burning, which is the dominant source of anthro-PPM_2.5_ emissions and is being increasingly adopted in Europe as a source of energy and heat. While the benefits of biomass burning in terms of greenhouse gas emissions are debatable, the significant negative impacts on air quality, health and biodiversity cannot be overlooked.

## Methods

### The Sherpa model

Sherpa^8,37–40^ belongs to the class of models known as integrated assessment models (IAM) which are tools used to simulate the potential changes in air pollutant concentration resulting from the application of measures to reduce emissions. The core of the Sherpa methodology relies on the definition of source-receptor relationships (SRR), that is, the relationships between changes in precursor emissions and pollutant concentrations. In other words, Sherpa is a simplified version of a CTM, based on algebric relationships linking PM_2.5_ concentration in each grid cell with gridded precursor emissions. The loss in accuracy resulting from the use of simplified SRRs rather than CTM simulations is minimal, whereas the gain in terms of the reduction in computing and human resources required to simulate the impact of emission scenarios results is substantial. This makes it possible to analyse a number of emission reduction scenarios in a very short period of time and makes IAMs an ideal tool to define air quality plans at the urban and regional levels.

Sherpa has been run with different CTMs^8^ but in recent years the JRC has used the model developed in the framework of the European monitoring and evaluation programme (EMEP) for Transboundary Long-Range Transported Air Pollutants as its default model. This CTM is also used as basis of the online Sherpa Cloud model (see Data availability section), which uses the same sample of cities as considered in the present paper. The EMEP model has been developed by the Meteorological Sythesizing Centre-West (MSC-W) in Oslo. The model is regularly updated and validated against observations^41^.

Sherpa relies on two main assumptions. The first is linearity between emissions and concentration changes, considering yearly averages. The second is that emissions and concentrations are spatially related by a ’bell-shape’ function. The two parameters that define the bell shape function are specific for each cell and precursor and are calculated on the basis of full CTM simulations (performed with 50% emission reductions over the whole model domain for each precursor). These two assumptions have been extensively tested by comparing Sherpa and the full CTM in various cities, regions and countries. The results of the validation process showed a good agreement between Sherpa and the CTM results for long term (i.e. yearly) PM_2.5_ averages as used in this work^42^.

The configuration of Sherpa used in this study is based on the EMEP v4.45 CTM^41^. The EMEP model domain covers the whole of Europe at 0.1° × 0.05° longitude-latitude spatial resolution (approximately 6 km in both latitude and longitude directions at middle latitudes).

Both Sherpa and the underlying EMEP model need emissions inventory data as input. In this study emissions by pollutant and sector are based on the Copernicus Atmospheric Monitoring Service (CAMS) v6.1 emission inventory from 2019. It is worth noting that a high-quality emissions inventory of good quality is key to obtaining good performance in CTM simulations and reliable SRRs. Unfortunately, uncertainties in emission inventories are still significant, in particular at the urban scale^43^. An analysis of the impact of the use of different inventories can be found elsewhere^44^.

It is important to point out that the CAMS emission inventory has recently been updated. The most important change is the inclusion of condensable gases in the estimates of PM_2.5_ emissions. Figure 11 (supplementary material) shows, for city cores and commuting zones, the change in the distribution density of residential sector emissions calculated using the CAMS inventory employed in this study (which includes condensable gases) and that obtained using the same CAMS inventory but without condensable gases. Emissions are markedly higher when condensable gases are included, reaching a 100% increase in some cities.

The current Sherpa version includes also an improved treatment of high-level and surface emissions by defining specific SRRs for the two types of sources. Two different SRRs are therefore provided as input to Sherpa, to account for the vertical split of the emissions.

The focus of this study is on urban areas and, therefore, the spatial resolution of the modelling tools is a key parameter. In this study the spatial resolution of both Sherpa and the underlying CTM is 0.1° × 0.05° (approximately 6 km). This spatial resolution represents a significant improvement compared to previous Sherpa versions and has been shown to be appropriate to capture urban background concentrations^45^. A comparison of the results obtained using Sherpa at two different spatial resolution (0.1° vs 0.05°) shows that the PM_2.5_ contributions of cities and the residential sector are slightly higher at fine-resolution runs^46^. This result suggests that the finer the spatial resolution, the better the capacity of the model to capture the actual characteristics of air pollution at the urban scale. However, it is likely that the findings of the Sherpa simulations in the smallest cities included in this study may be more subject to uncertainty, because these cities cover only a few grid cells.

Meteorological input data from the integrated forecasting system of the European Medium Range Weather Forecasts (ECMWF) date to 2015. Meteorological files are retrieved at 0.1° × 0.1° spatial resolution and downscaled to 0.1° × 0.05°. The same meteorological data are used for Sherpa and EMEP simulations. We chose 2015 has the reference year for consistency with previous Sherpa applications.

Spatial-sector-pollutant source allocations are calculated for the grid cell within each city that shows the highest PM_2.5_ concentration. This choice is in line with EU Directive 2008/50/EC, which states that air quality plans must be developed wherever exceedances are identified within an air quality zone.

### Cities and functional urban areas

Cities and FUAs were defined in accordance with the definition provided by the OECD^47,48^. Cities are local administrative units (or aggregates of contiguous local administrative units) with a population of 50,000 or more and a population density of more than 1500 inhabitants/km^2^. FUAs consist of the core city plus its commuting zone, that is, surrounding local administrative units where at least 15% of residents work in the city core. The total population of cities and FUAs included in the study accounts for, respectively, about 40% and 64% of total population of the study area. Core and FUA boundaries are regularly updated and made available in the framework of the ’Urban audit’ project jointly promoted by the EU Commission and OECD^49^.

### Supplementary Information

Supplementary Figures.

## Data Availability

The source allocation data for the 708 cities produced by the European Commission (EC-JRC) can be accessed at: https://data.jrc.ec.europa.eu/dataset/ac97b944-2635-4122-8c05-4f0d0cdc8644. Data can also be accessed and visualized via the Sherpa-Cloud web application available at this link: https://jeodpp.jrc.ec.europa.eu/eu/dashboard/voila/render/SHERPA/Sherpa.ipynb. The access to the application requires a EUlogin account, the European Commission’s user authentication service (https://wikis.ec.europa.eu/display/NAITDOC/EU+Login+-+European+Commission+Authentication+Service).

## Code availability

Codes for calculation and data processing are written in R and are available from the correspond- ing author upon request. The Python code to run the Sherpa model is freely available at GitHub: https://github.com/enricopisoni/SHERPA-simulation.
