# Supplementary material for: Reducing biomass burning is key to decrease PM2.5 exposure in European cities
Source: Sci Rep. 2024 May 3;14:10210. doi: 10.1038/s41598-024-60946-2 (PMC11068762; doi:10.1038/s41598-024-60946-2)
Supplement: Supplementary file 1 — Supplementary Figures. [file 41598_2024_60946_MOESM1_ESM.docx]

# Supplementary material


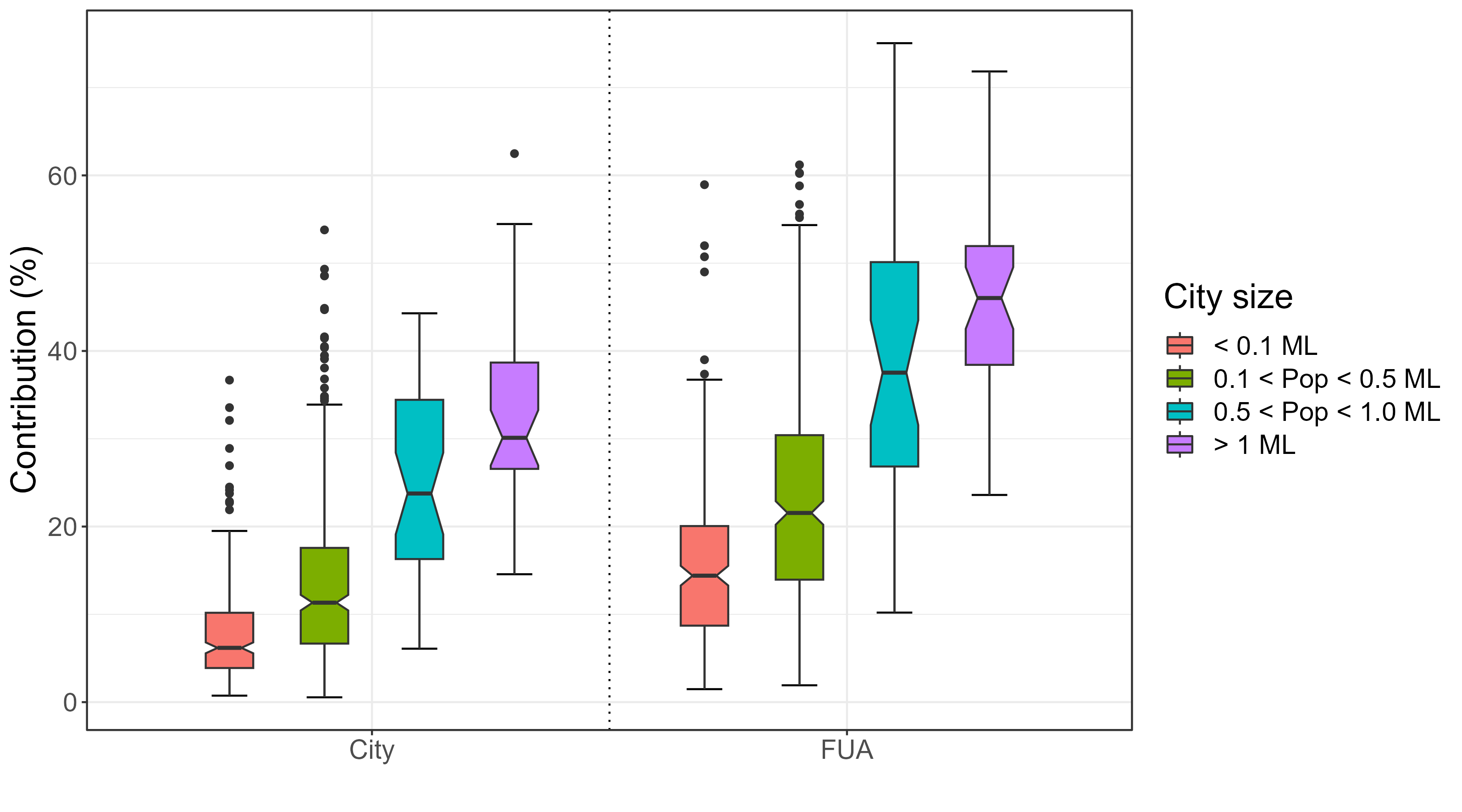


Figure 7: **City and FUA contribution to local PM**2*.*5 **grouped by city size.** Boxplot diagrams summarizing city and FUA contribution to PM2*.*5 urban concentration, grouped by four city size classes.


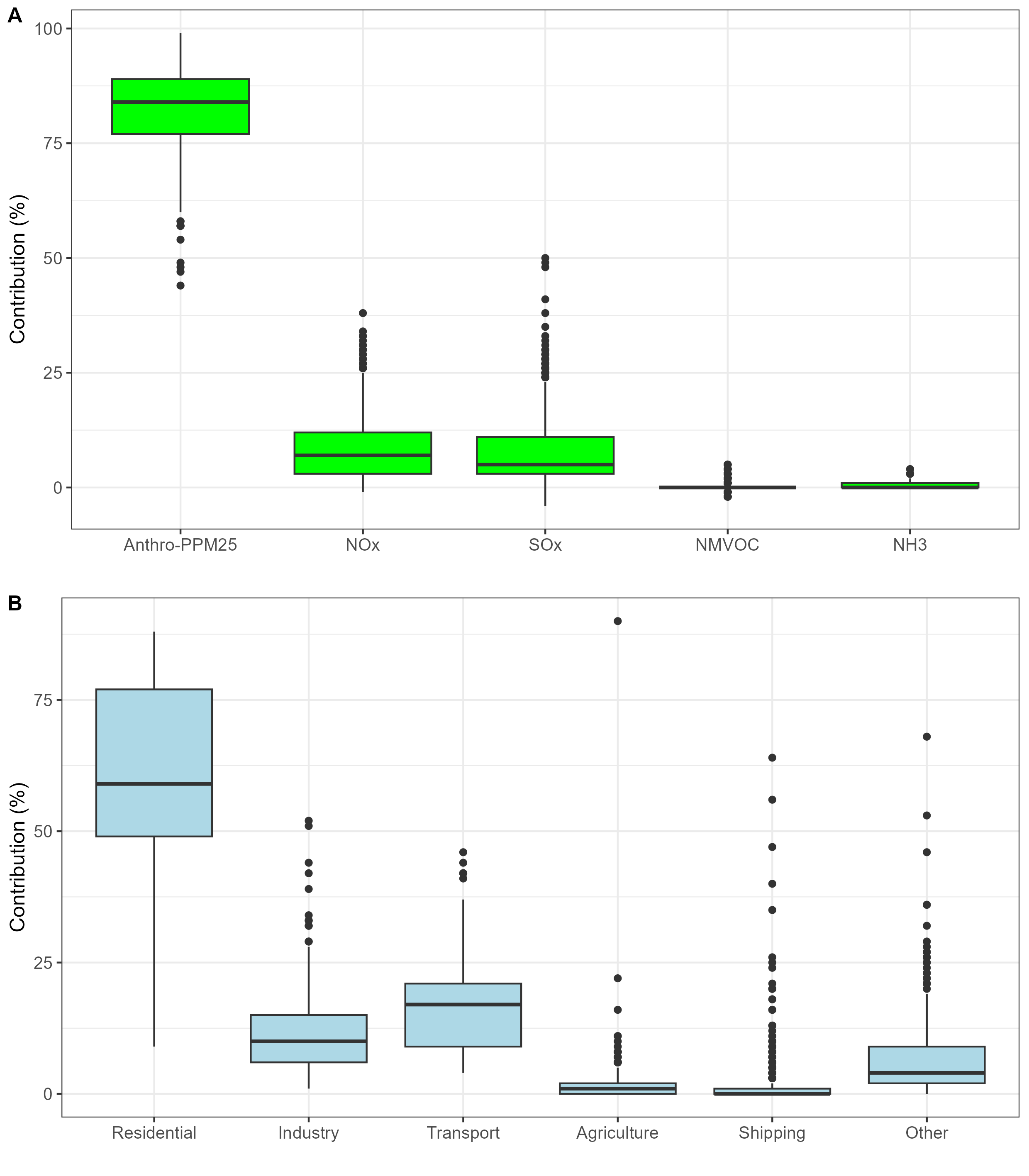


Figure 8: **Residential contribution and anthro-PPM**2*.*5**.** Boxplot of the contribution of each precursor to the total residential contribution to PM2*.*5 (panel A) and of the single source sectors to primary anthropogenic PM2*.*5 (anthro-PPM2*.*5) (panel B).


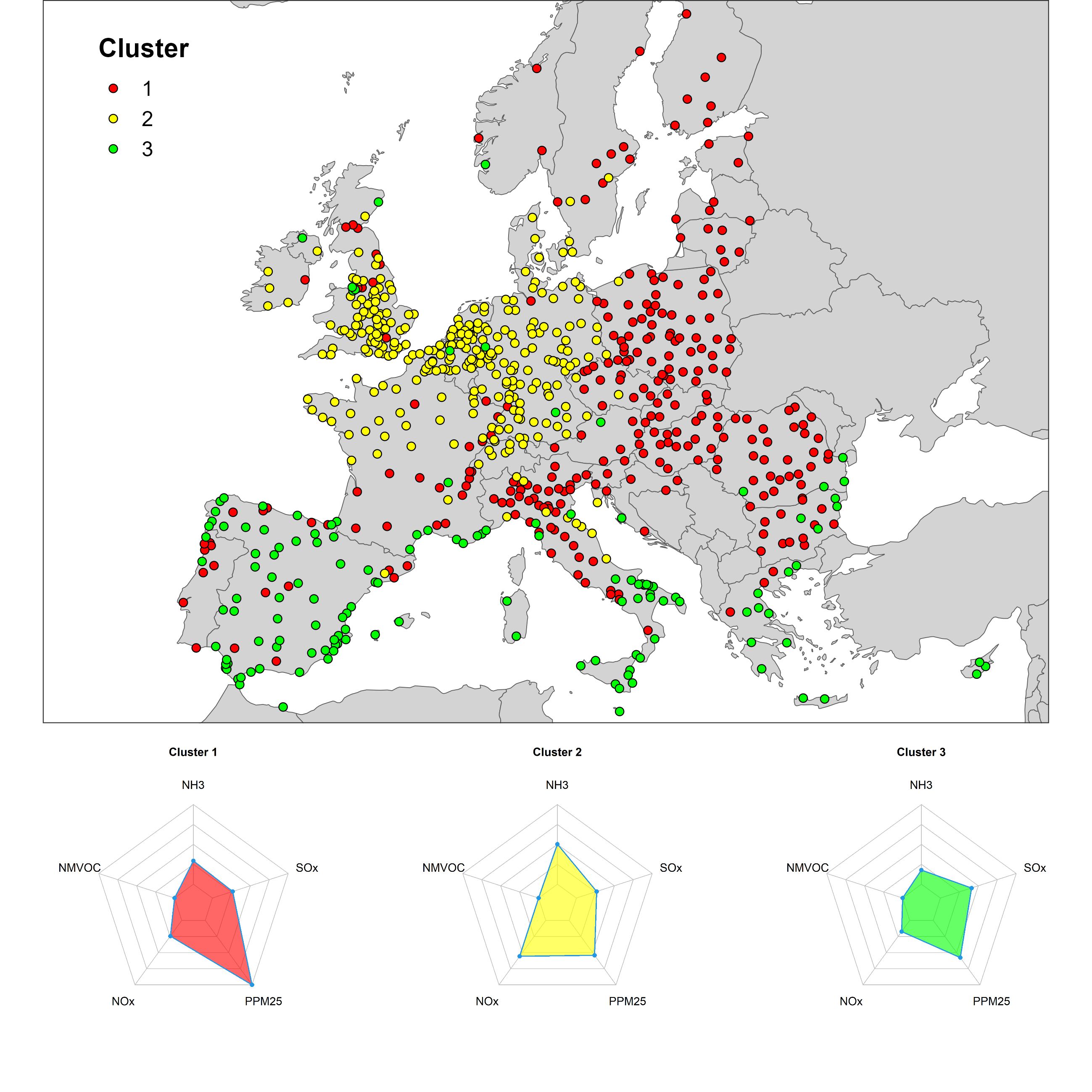


Figure 9: **Clusters of cities by precursors.** Map of cities clusterized on the basis of precursor emissions percentage contributions to PM2*.*5 concentrations (upper panel) and diagrams of the weight factors associated with each cluster (lower panels).


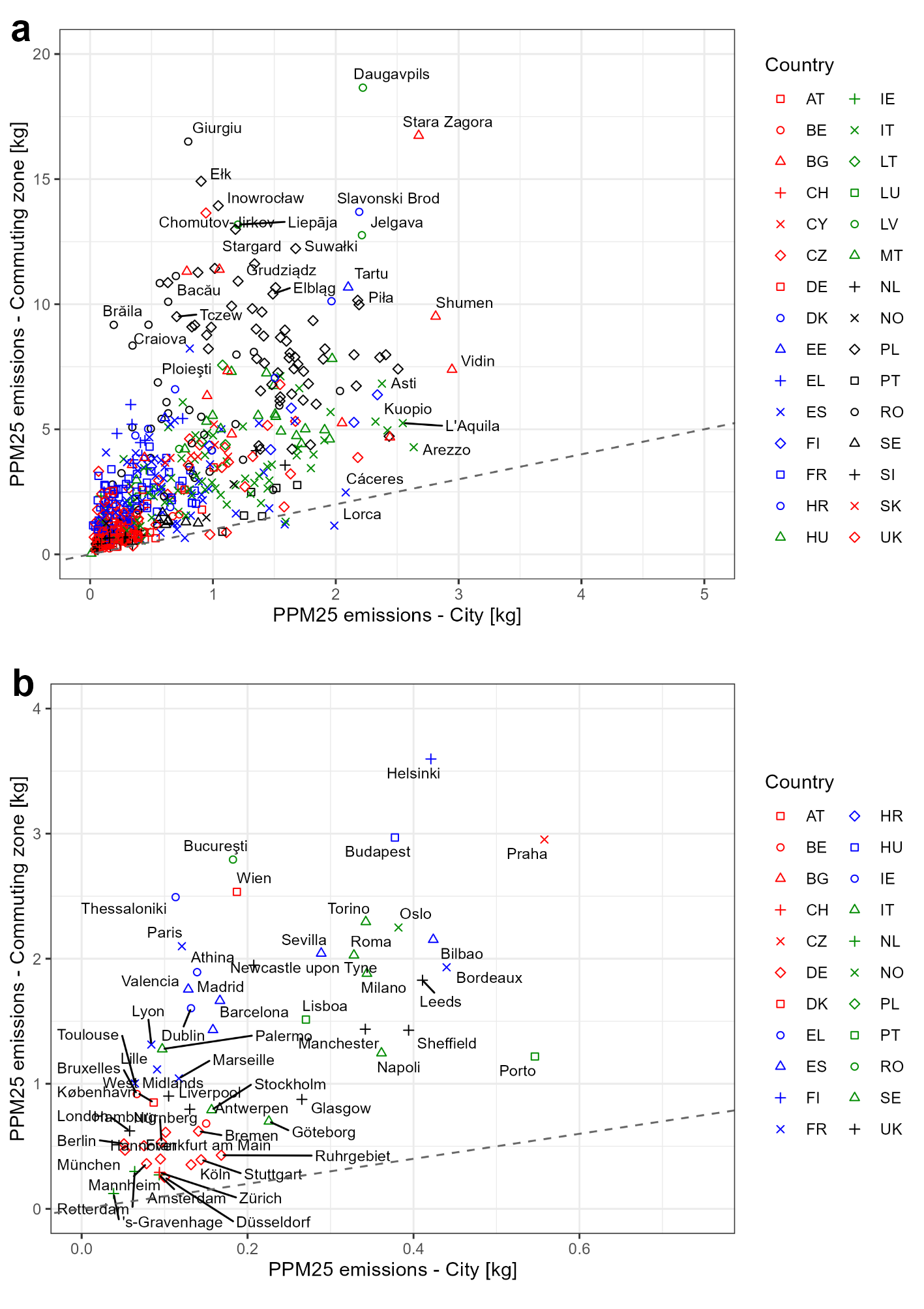


Figure 10: **Per capita emissions of anthro-PPM**2*.*5 **from the residential sector.** The Figure shows per capita emissions of primary anthropogenic PM2*.*5 (anthro-PPM2*.*5) from the residential sector in cities (x-axis) and commuting areas (y-axis). Panel a) includes all cities while panel b) includes only cities with FUA population greater than 1 Ml.


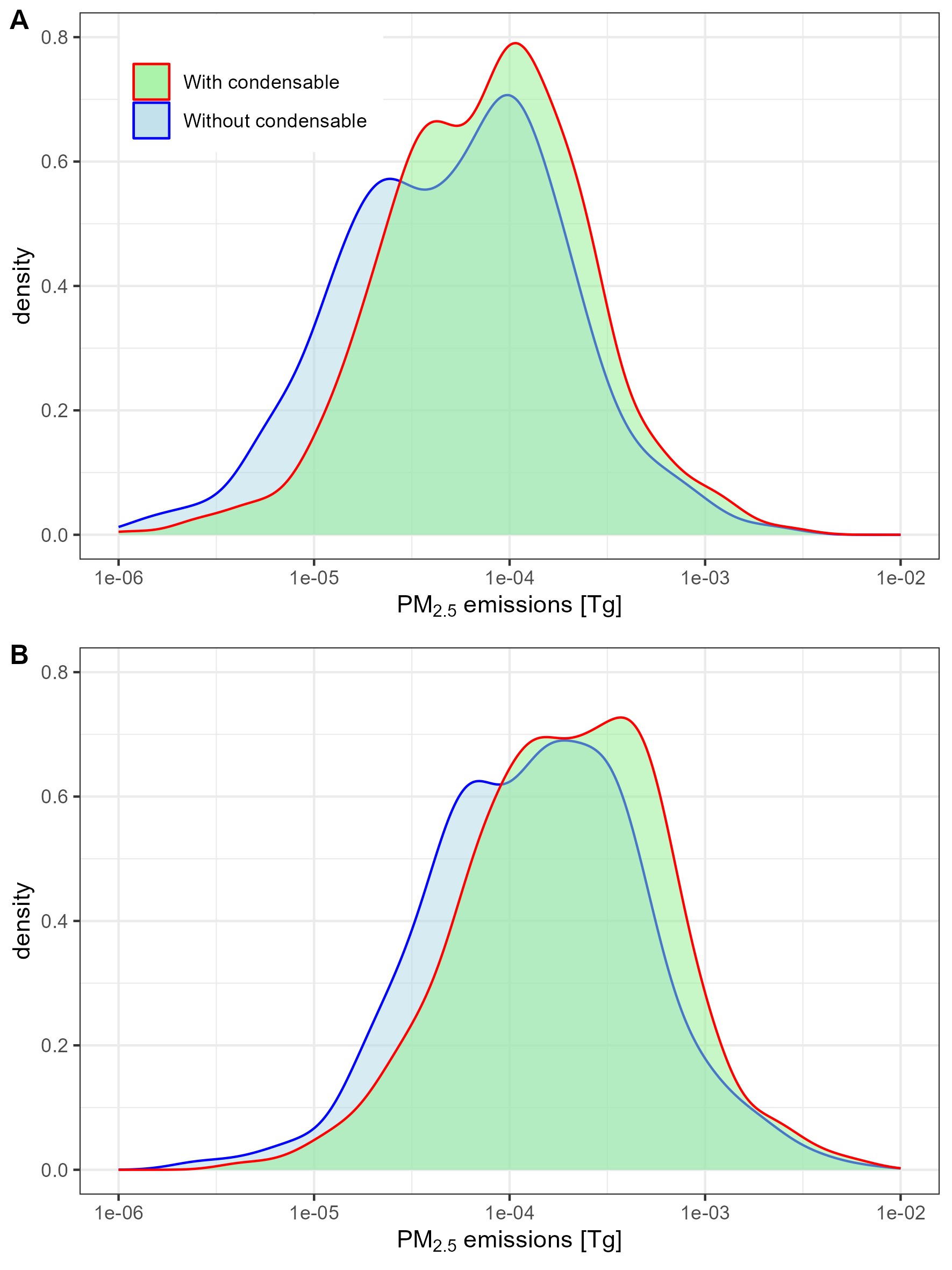


Figure 11: **Primary anthropogenic PM**2*.*5 **(anthro-PPM**2*.*5**) emissions of the residential sector with and without the inclusion of condensable gases.** Probability density distribution of city core (panel A) and commuting zone (panel B) emissions of primary anthropogenic PM2*.*5 (anthro-PPM2*.*5) from the residential sector with and without the inclusion of condensable gases.


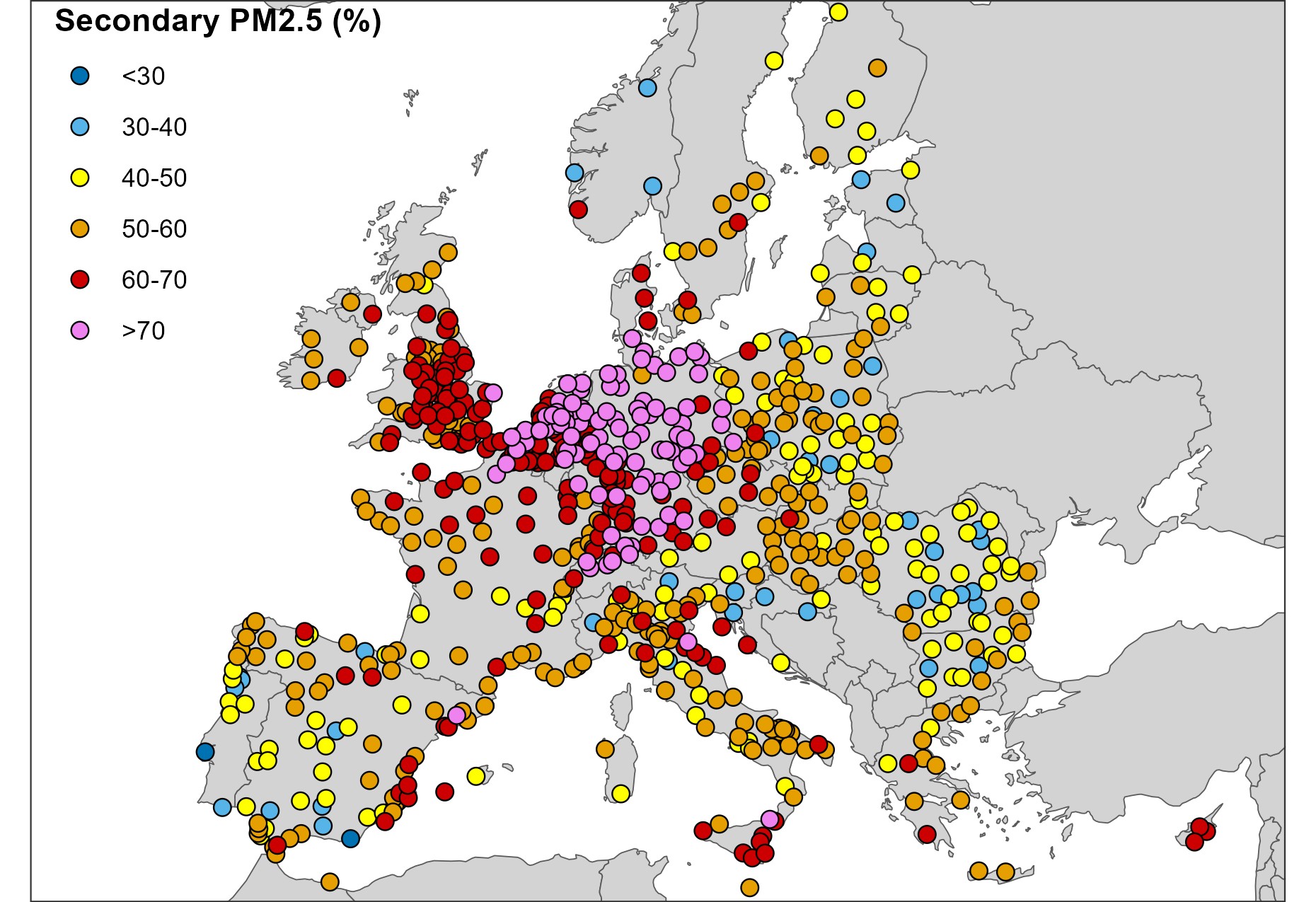


Figure 12: **Secondary PM**2*.*5 **compared with total PM**2*.*5**.** Values are shown as a percentage of total PM2*.*5 mass in each city.


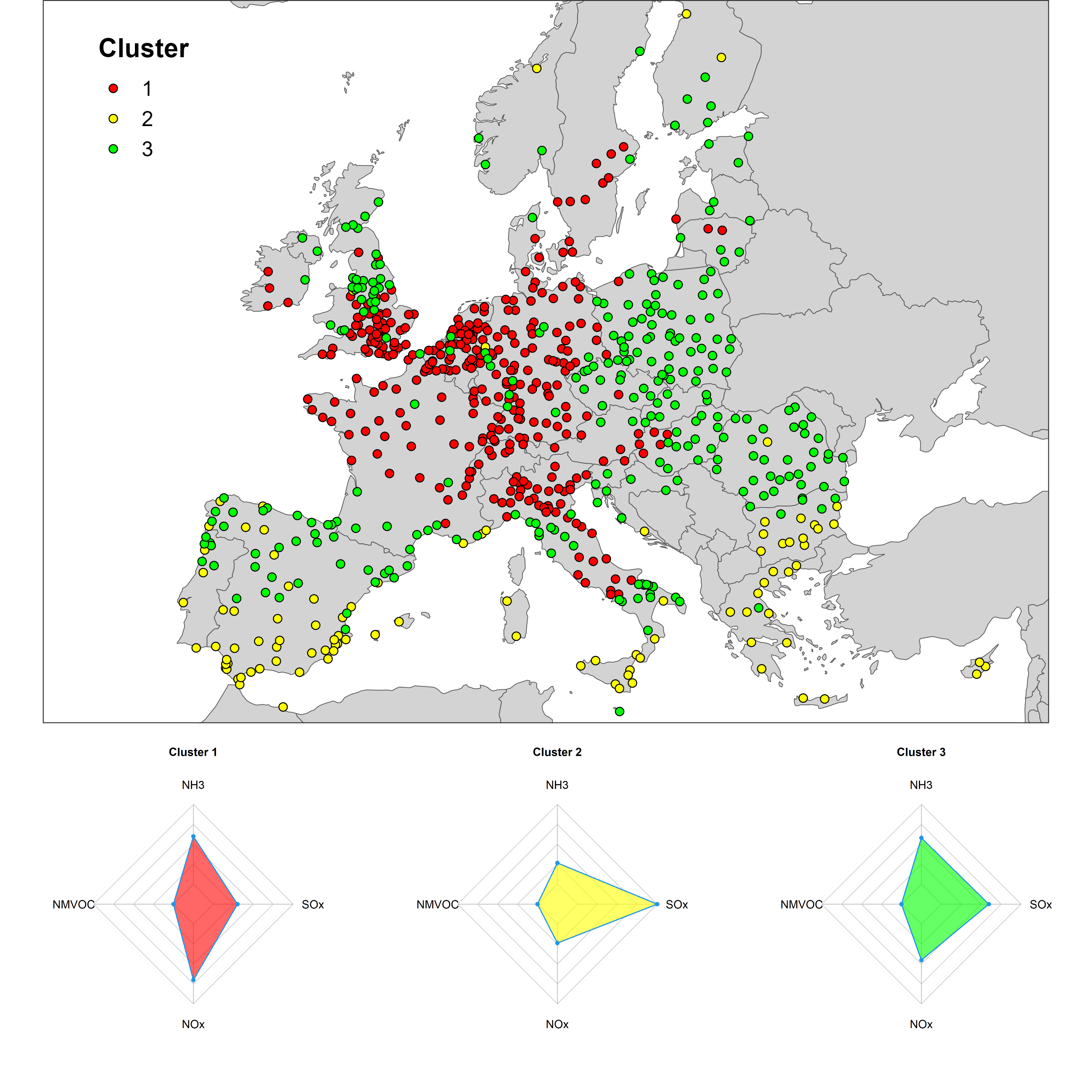


Figure 13: **Clusters of cities by precursors of secondary PM**2*.*5**.** Map of cities clustered on the basis of percentage contributions to PM2*.*5 concentrations (upper panel) and diagrams of the weight factors associated with each cluster (lower panels).


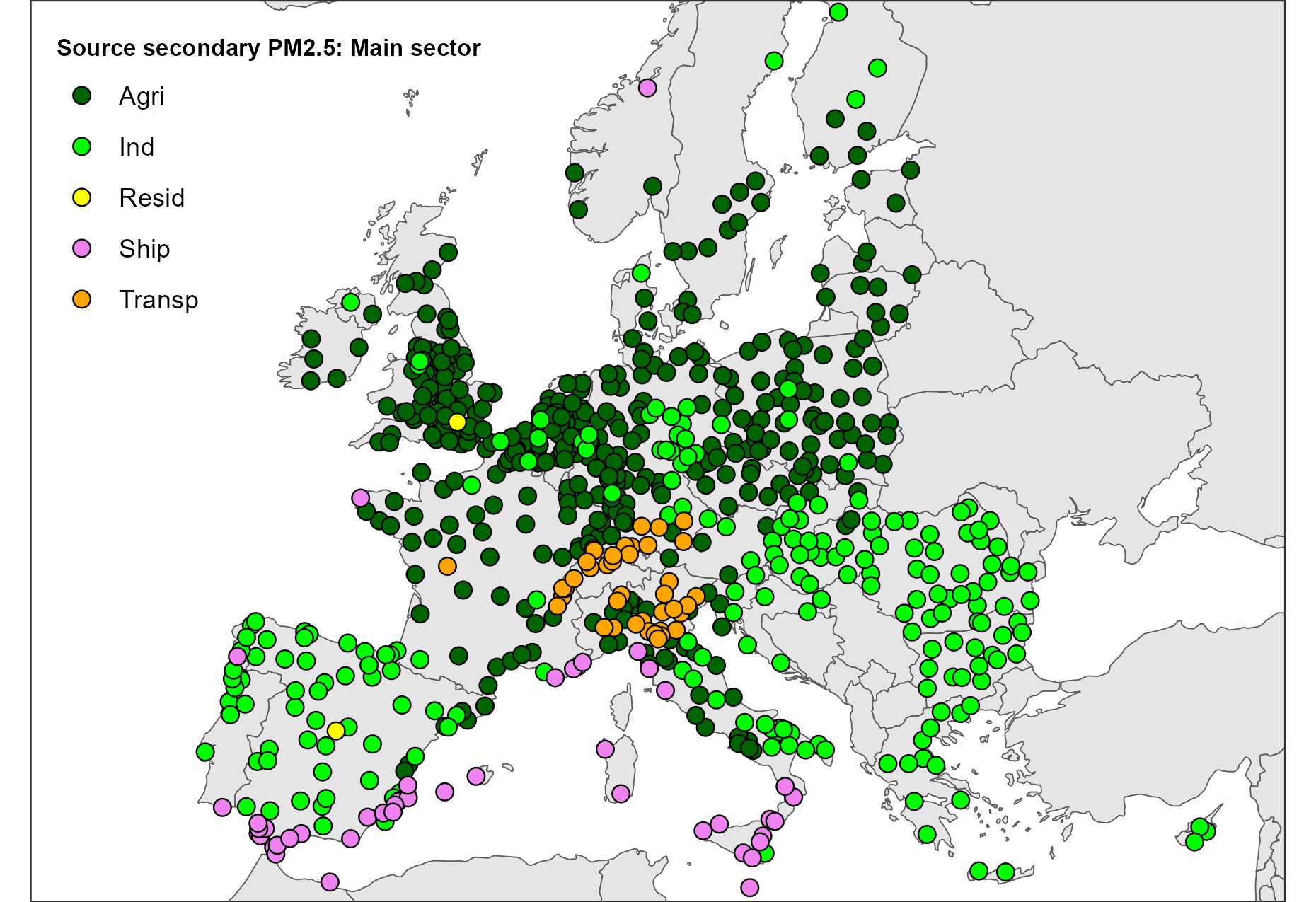


Figure 14: **Main source sector of secondary PM**2*.*5**.** Each point representing a city.


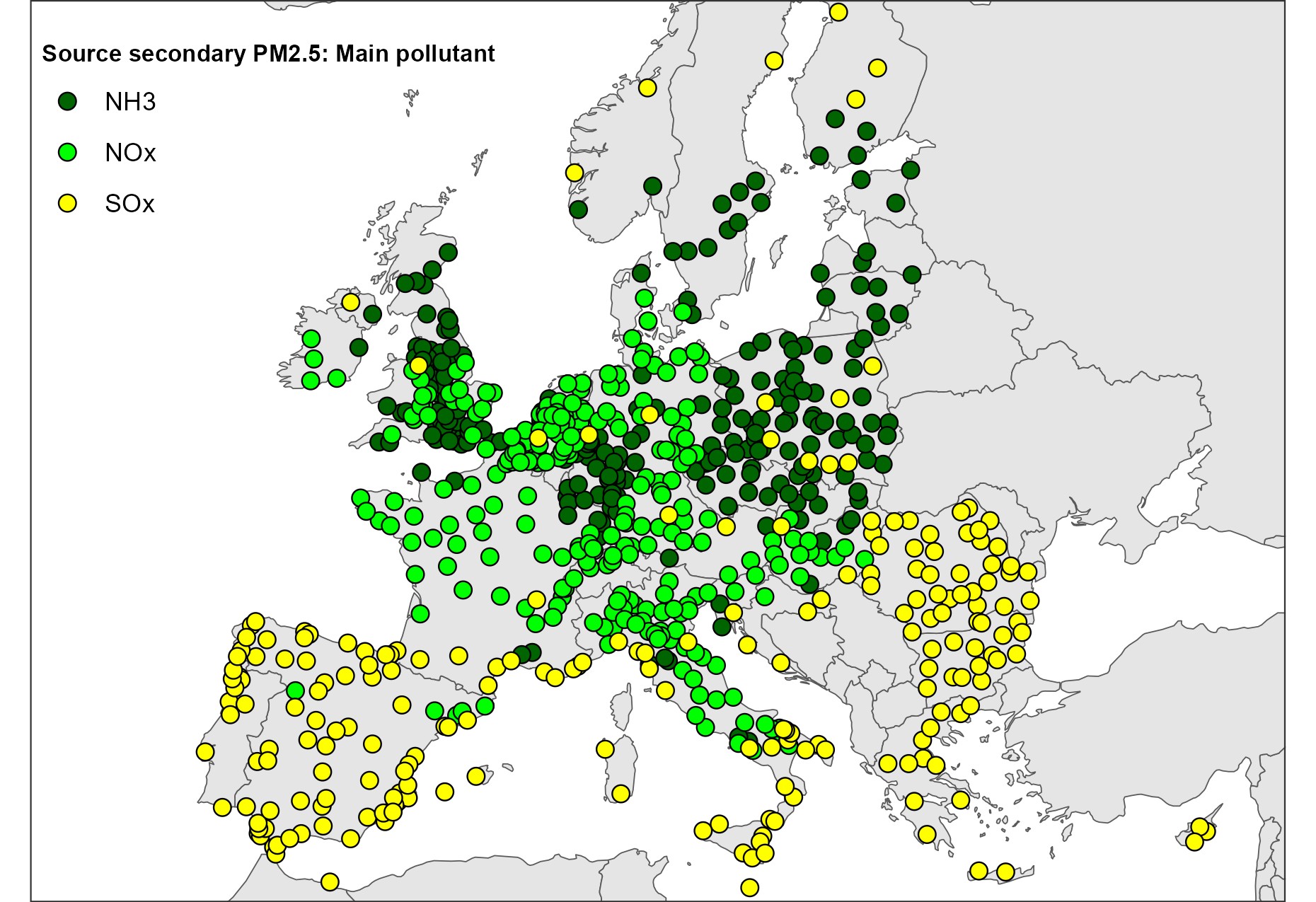


Figure 15: **Main precursor of secondary PM**2*.*5**.** Each point representing a city.
